# A Conversion Formula for Comparing Pulse Oximeter Desaturation Rates Obtained with Different Averaging Times

**DOI:** 10.1371/journal.pone.0087280

**Published:** 2014-01-28

**Authors:** Jan Vagedes, Anja Bialkowski, Cornelia Wiechers, Christian F. Poets, Klaus Dietz

**Affiliations:** 1 Children's Hospital, Department of Neonatology, University of Tübingen, Tübingen, Germany; 2 ARCIM-Institute, Research Department, Filderklinik, Filderstadt, Germany; 3 Department of Medical Biometry, University of Tübingen, Tübingen, Germany; University of Louisville, United States of America

## Abstract

**Objective:**

The number of desaturations determined in recordings of pulse oximeter saturation (SpO_2_) primarily depends on the time over which values are averaged. As the averaging time in pulse oximeters is not standardized, it varies considerably between centers. To make SpO_2_ data comparable, it is thus desirable to have a formula that allows conversion between desaturation rates obtained using different averaging times for various desaturation levels and minimal durations.

**Methods:**

Oxygen saturation was measured for 170 hours in 12 preterm infants with a mean number of 65 desaturations <90% per hour of arbitrary duration by using a pulse oximeter in a 2–4 s averaging mode. Using 7 different averaging times between 3 and 16 seconds, the raw red-to-infrared data were reprocessed to determine the number of desaturations (D). The whole procedure was carried out for 7 different minimal desaturation durations (≥1, ≥5, ≥10, ≥15, ≥20, ≥25, ≥30 s) below SpO_2_ threshold values of 80%, 85% or 90% to finally reach a conversion formula. The formula was validated by splitting the infants into two groups of six children each and using one group each as a training set and the other one as a test set.

**Results:**

Based on the linear relationship found between the logarithm of the desaturation rate and the logarithm of the averaging time, the conversion formula is: D_2_ = D_1_ (T_2_/T_1_)^c^, where D_2_ is the desaturation rate for the desired averaging time T_2_, and D_1_ is the desaturation rate for the original averaging time T_1_, with the exponent c depending on the desaturation threshold and the minimal desaturation duration. The median error when applying this formula was 2.6%.

**Conclusion:**

This formula enables the conversion of desaturation rates between different averaging times for various desaturation thresholds and minimal desaturation durations.

## Introduction

Many clinical management decisions in intensive care, sleep medicine and neonatology are based on a patient's SpO_2_, the type and number of desaturation events that he/she experiences as well as on the time spent within different saturation ranges [1], [2].

The advantages of pulse oximetry include its ease of use and close correlation with arterial oxygen saturation, at least above 70% SpO_2_, which allows for a substantial reduction in arterial blood gas measurements. Limitations are its susceptibility to motion [3], [4], low perfusion [5], [6], skin pigmentation [7], ambient light [8], electromagnetic radiation or nonfunctional hemoglobins (carboxyhemoglobin or methemoglobin). In the case of motion-induced artefacts, new generation pulse oximeters employ methods such as plethysmographic waveform analysis to filter the “noise”, or use other approaches such as longer averaging times. In contrast to the effects of motion, the influence of the averaging time on desaturation levels, durations and extent is often underestimated or not mentioned [9]–[16].

In terms of measurement accuracy, beat-to-beat measurements would be desirable. The beat-to-beat mode has the highest possible resolution for SpO_2_ measurements. With every pulse beat, based on the absorption of red and infrared light, the oxygen saturation is calculated. Especially in neonatology, where patients often have fast changes in oxygen saturation, the beat-to-beat modus helps to identify all changes in oxygen saturation actually occurring, but at the expense of a high monitor alarm rate, potentially desensitizing nursing staff. For research purposes, however, it continues to yield the most detailed level of information about the stability of a patient's oxygenation, and much of the reference data available in infants has been established using the beat-to-beat mode [17]–[23]. However, as every shaky signal raises the probability of falsely low values, which would result in constant alarms if measured in a beat-to-beat mode, modern oximeters come with an adjustable averaging time, but the resultant smoothing of the SpO_2_ value curve leads to an increased risk of missed desaturations.

Furthermore, as soon as the desaturation duration is taken into account, the dependency of the desaturation rate from the averaging time varies: With short desaturation durations (<10 s) the desaturation rate decreases with increasing averaging times, and vice versa, with long desaturation durations (≥20 s) the desaturation rate increases with increasing averaging times [24].

Only a few studies have compared desaturation rates measured with different averaging times [25]–[31]. We have recently demonstrated that the SpO_2_ nadir, and the minimal desaturation duration and extent, depend significantly on the averaging time used [24]. We compared recordings obtained with the shortest vs. the longest averaging time available on an instrument within the same patient. We found that the number of desaturations to <80% of arbitrary desaturation duration varied almost 6-fold, when an averaging time of 3 s was used instead of 16 s, that the SpO_2_ nadir was significantly lower with the shorter averaging time, and that the maximum duration of individual desaturation episodes was longer when using a longer averaging time. Having verified the criticality of the averaging time used, a new problem arose: how can we compare the results obtained when using different averaging times?

To the best of our knowledge, no method has yet been put forward for comparing SpO_2_ measurements obtained from infants when using different averaging times. Thus, the objective of the present study was to generate a formula that allows conversion between desaturation rates obtained using different averaging times for various desaturation levels and minimal durations; this has involved re-analysing the data from our previous study[24].

## Methods

### Ethics Statement

The study protocol was approved by the Tübingen University Hospital Ethics Committee and parents had given written, informed consent.

Fifteen spontaneously breathing infants admitted to the Department of Neonatology at Tübingen University Hospital were originally included. Inclusion criteria were the occurrence of recurrent desaturations due to apnea of prematurity in otherwise healthy infants.

In contrast to other studies that analyze the effect of averaging on SpO_2_, our data are based on the same red-to-infrared signal; this was made possible by the subsequent reprocessing of the original raw red-to-infrared light absorption data. The reprocessing was carried out for every patient with the help of the oximeter manufacturer. Using this method, we obtained different SpO_2_ values for the same patient by reprocessing the same raw red-to-infrared absorption data with 7 different averaging times (3, 5, 8,10,12,14,16 s) below SpO_2_ threshold values of 80%, 85% or 90%. The manufacturer uses an algorithm with a moving average for short averaging times (2–4 s and 4–6 s). We thus assumed a 3 or 5 s averaging time. In the following, the reprocessed data will be called “observed” values, because the SpO_2_ values are based on the same algorithms an oximeter uses to calculate the SpO_2_ values based on the raw red-to-infrared absorption data. The manufacturer claims that, based on this approach, all their software is validated against measured values.

The data were screened for good signal quality, as recommended (i.e., a Signal-IQ >30) [32]–[35]. We identified desaturations, defined as SpO_2_ below 80%, 85%, or 90%, and with minimum durations of ≥1, ≥5, ≥10, ≥15, ≥20, ≥25, ≥30 s, respectively, from recordings of SpO_2_ using varying averaging times.

The relationship between desaturation rate and averaging time was analyzed using JMP statistical software (SAS institutes Inc., USA) for the 3 desaturation thresholds, 7 different minimal durations and 7 different averaging times mentioned above. This was carried out on all periods without missing observations. After having found a formula including an exponent c in dependency of the desaturation threshold and the minimal desaturation duration, we calculated the exponents c for each infant for all 21 combinations (three thresholds and seven minimal desaturation durations) and divided the infants into two groups.

Assignment to Groups 1 and 2 was based on the mean conversion formula exponent c of each infant, sorted in ascending order, with infants then being randomly assigned to either group. The aim of dividing the infants into two groups was to split the infants into a training set and a test set: We calculated desaturation rates (predicted values) for different averaging times, desaturation durations and SpO_2_ thresholds for the Group 2 dataset, based on the formula generated with the Group 1 dataset, and validated this formula by analysing the correlation between predicted and observed values for Group 2. The same was carried out in reverse by calculating values (predicted values) for Group 1 based on the Group 2 dataset, which was then tested in the same manner with the Group 1 dataset (concordance correlation [36] between predicted values for Group 1 and observed values of Group 1). Analogous to the criteria of Bland and Altman the mean of paired values is taken into consideration when using the concordance correlation by calculating a scale shift (quotient of the two standard deviations) and a location shift (standardized differences of the paired values). A concordance correlation >0.95 has been defined as “substantial”, one >0.99 as “almost perfect”[37].

## Results

The fifteen infants had a gestational age between 32 and 33 weeks at study (24 and 27 weeks at birth). Three infants were excluded because they did not have desaturations below all three thresholds and with all averaging times and maximum desaturation duration.

217 hours of recorded SpO_2_ values were recorded. Of these, 22% (47 hours) were excluded due to missing observations or because the observation interval had started or ended with a desaturation (taking into account the three thresholds, the seven desaturation durations and seven averaging times); this left 170 hours from 12 infants (mean: 14.1 hours/per infant, min: 6.6 hours, max: 24.0 hours) remaining for analysis. These remaining duration of recording ensured that the number of events for all conditions were positive (i.e., >0), such that a conversion formula was applicable. The excluded hours were not usable for a conversion formula due to missing observations.

### Conversion formula

We found a linear relationship between the logarithm of the desaturation rate per hour and the logarithm of the averaging time; we used log to base 10. The slope of the linear regression was positive for a minimal duration of 20 or 30 s, and negative for a minimal desaturation duration of 1 or 5 s, which means that the slope depended on the desaturation duration, which has been discussed in our previous publication [24].

Based on this linear relationship, the conversion formula was found to be:
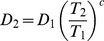



In this formula D_2_ is the desaturation rate for the desired averaging time T_2_, and D_1_ is the desaturation rate for the original averaging time T_1_. The exponent c depends on the desaturation threshold and the minimal desaturation duration and was obtained for all 21 combinations: 3 desaturation thresholds (80%, 85%, 90%) and 7 desaturation durations for every infant. As described above, the infants' mean exponents were sorted in ascending order and infants then randomly assigned to Group 1 or 2. After this allocation of each child to one of the two groups, the exponents were re-estimated for the 21 conditions (see Table 1).

**Table 1:** Exponents c for the conversion formula D_2_ = D_1_ (T_2_/T_1_)^c^ for 3 different SpO2 thresholds, seven different minimal desaturation durations and for two different infant groups.

SpO_2_	Minimal desaturation	Exponent c (95% CI)	
Threshold (%)	duration (sec)	Group 1	Group 2
**90**	1	−0.918 (−0.945; −0.891)	−0.834 (−0.854; −0.814)
	5	−0.459 (−0.502; −0.416)	−0.409 (−0.451; −0.367)
	10	−0.110 (−0.120; −0.101)	−0.104 (−0.118; −0.089)
	15	−0.016 (−0.027; −0.004)	−0.024 (−0.034; −0.014)
	20	0.067 (0.058; 0.077)	0.033 (0.028; 0.038)
	25	0.121 (0.109; 0.133)	0.083 (0.075; 0.092)
	30	0.150 (0.136; 0.165)	0.108 (0.099; 0.116)
**85**	1	−1.000 (−1.045; −0.954)	−0.904 (−0.933; −0.875)
	5	−0.485 (−0.547; −0.423)	−0.399 (−0.445; −0.352)
	10	−0.057 (−0.075; −0.038)	−0.046 (−0.057; −0.034)
	15	0.079 (0.060; 0.099)	0.034 (0.018; 0.050)
	20	0.193 (0.178; 0.209)	0.141 (0.123; 0.158)
	25	0.326 (0.292; 0.360)	0.195 (0.190; 0.201)
	30	0.375 (0.334; 0.415)	0.235 (0.217; 0.253)
**80**	1	−1.119 (−1.178; −1.060)	−1.037 (−1.082; −0.993)
	5	−0.482 (−0.547; −0.417)	−0.462 (−0.520; −0.404)
	10	−0.074 (−0.103; −0.045)	−0.109 (−0.132; −0.086)
	15	0.060 (0.031; 0.089)	0.020 (0.000; 0.041)
	20	0.121 (0.067; 0.175)	0.193 (0.169; 0.218)
	25	0.228 (0.181; 0.275)	0.188 (0.144; 0.233)
	30	0.318 (0.257; 0.379)	0.347 (0.321; 0.374)

There are 9 statistically significant differences in the exponents between the two groups after adjustment for multiple testing according to Bonferroni-Holm, but these are not relevant as shown in the following comparison of observed with predicted values.

**Figure 1:**
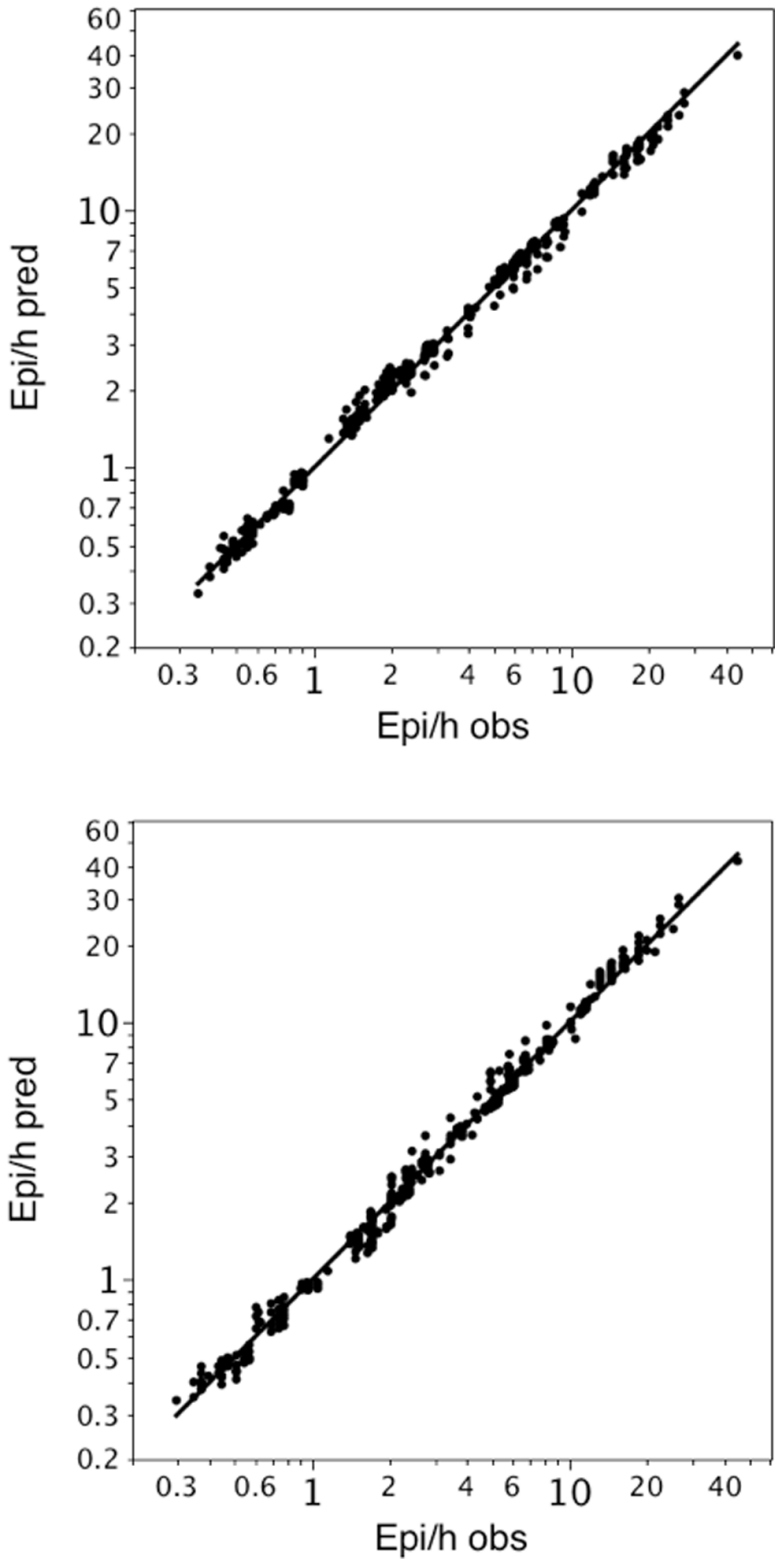
a. Bivariate fit of observed and predicted desaturations per hour with Group 1 as training set. Group 1 has been used as training set to generate the different exponents c for the formula. For group 2 (test set) predicted desaturations have been calculated (based on the exponents of group 1). The predicted desaturations (Npred/h) for Group 2 are plotted against the observed desaturations (Nobs/h) of Group 2. The concordance correlation coefficient was 0.997 b. Bivariate fit of observed and predicted desaturations per hour with Group 2 as training set. Group 2 has been used as training and Group 1 as test set. The predicted desaturations (Npred/h) for Group 1 are plotted against the observed desaturations (Nobs/h) of Group 1. The concordance correlation coefficient was 0.998

We demonstrate the precision of the conversion formula by plotting the observed (Nobs/h) against the predicted (Npred/h) desaturation rates for the three different SpO_2_-thresholds, 7 minimal durations and 7 averaging times. The agreement between training and test set is shown in Figure 1a and 1b.

If Group 1 was chosen as the training set and Group 2 as the test set, the median percentage error was 3.9%. If Group 2 was chosen as the training set and Group 1 as the test set, the median percentage error was 4.6%.

The values for the concordance correlation coefficients were 0.9966 (95 CI: 0.9961; 0.9970) if Group 1 was the training and Group 2 the test set and 0.9976 (95 CI: 0.9972; 0.9979) if Group 2 was the training and Group 1 the test set.

Having shown high agreements, low median percentage errors between predicted and observed values for both groups and almost perfect concordance correlation coefficients, we calculated the exponents c for the entire dataset of 12 infants (Groups 1? Table 2).

**Table 2:** Exponents c for the conversion formula D_2_ = D_1_ (T_2_/T_1_)^c^ for 3 different SpO2 thresholds, seven different minimal desaturation durations and for all 12 infants (Group 1 & 2).

SpO_2_	Minimal desaturation	Exponent c (95% CI)
Threshold (%)	duration (sec)	Group 1 & 2
**90**	1	−0.876 (−0.898; −0.854)
	5	−0.433 (−0.476; −0.391)
	10	−0.107 (−0.118; −0.096)
	15	−0.020 (−0.028; −0.011)
	20	0.050 (0.045; 0.055)
	25	0.102 (0.093; 0.111)
	30	0.129 (0.118; 0.139)
**85**	1	−0.954 (−0.991; −0.916)
	5	−0.442 (−0.497; −0.388)
	10	−0.051 (−0.065; −0.037)
	15	0.056 (0.044; 0.069)
	20	0.167 (0.156; 0.178)
	25	0.260 (0.242; 0.277)
	30	0.306 (0.287; 0.324)
**80**	1	−1.081 (−1.133; −1.029)
	5	−0.473 (−0.534; −0.411)
	10	−0.091 (−0.115; −0.066)
	15	0.042 (0.021; 0.063)
	20	0.156 (0.122; 0.191)
	25	0.209 (0.171; 0.246)
	30	0.333 (0.308; 0.357)

To make the conversion process straightforward, as required for daily clinical use, Table 3 gives an example for desaturations of arbitrary duration and SpO_2_ <80%. For example, if 10 events/h had been counted with an averaging time of 16 s, this number would have to be multiplied by 6.108 to get the number of severe events with an averaging time of 3 s. In this case one would expect about 61 events with an averaging time of 3 s.

**Table 3:** Conversion factors between different averaging times for events (SpO_2_ <80%) of arbitrary duration.

		Tobs (sec)						
		3	5	8	10	12	14	16
**Tdes (sec)**	**3**	1.000	1.737	2.887	3.675	4.475	5.287	6.108
	**5**	0.576	1.000	1.662	2.116	2.576	3.044	3.516
	**8**	0.346	0.602	1.000	1.273	1.550	1.831	2.116
	**10**	0.272	0.473	0.786	1.000	1.218	1.439	1.662
	**12**	0.223	0.388	0.645	0.821	1.000	1.181	1.365
	**14**	0.189	0.329	0.546	0.695	0.847	1.000	1.155
	**16**	0.164	0.284	0.473	0.602	0.733	0.866	1.000

Tobs is the observed and Tdes the desired averaging time (s). For example, if 5 events/h had been counted with an averaging time of 16 s (Tobs), this number would have to be multiplied by 6.108 to get the number of severe events with an averaging time of 3 s (Tdes). In this case one would expect about 31 events with an averaging time of 3 s.

## Discussion

In recent years, much has been invested to increase signal quality in pulse oximetry, including pattern analysis of the pulse waveform. The clinical relevance of the averaging time, however, has received little attention. After verifying the extent of the influence the averaging time has on desaturation levels, minimal duration and extent [24], we now focussed our attention on generating a formula by which the number of desaturations measured with one averaging time can be extrapolated to the number of events measured with a different averaging time. This is important to make data on a subject's desaturation rate obtained with a specific averaging time comparable to data sampled with another averaging time.

To the best of our knowledge, such a formula has not yet been published. We took into account different desaturation durations and thresholds. It turned out that there is a linear relationship between the logarithm of the desaturation rate and the logarithm of the averaging time and, moreover, that the slope of the regression line depends on the desaturation duration and the threshold value.

**Figure 2:**
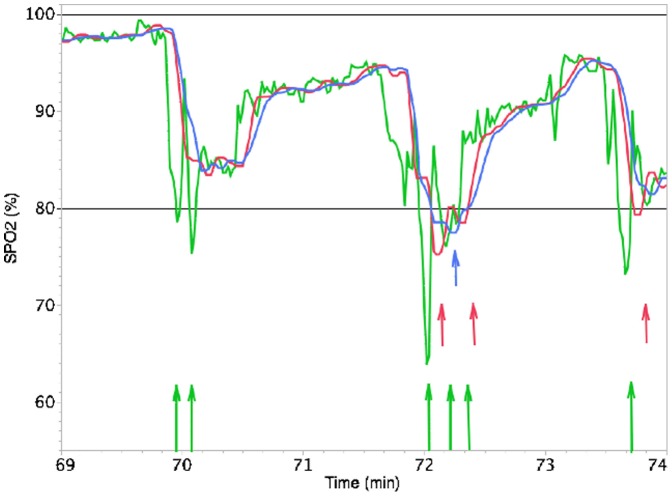
Influence of the averaging time and the desaturation duration on the number of desaturations. SpO_2_ recordings for averaging times of 3 seconds (green), 10 seconds (red) and 16 seconds (blue). For an alarm threshold at 80% SpO_2_ (straight line) and an arbitrary desaturation duration, an averaging time of 3 s results in 6 desaturations, while an averaging time of 10 or 16 s results in 3 and 1 desaturation(s), respectively. For a desaturation duration ≥15 s, an averaging time of 3 s or 10 s results in no desaturation, while an averaging time of 16 s results in 1 desaturation.

For a minimal desaturation event duration of 1 or 5 s, the slope was negative, which means, that fewer desaturations with a duration of 1 or 5 s are counted when a longer averaging time is used. This might be explained by the fact that several short desaturations are summed up to one long desaturation by smoothing the SpO_2_ curve as soon as longer averaging times are used (Fig. 2).

As shown in our previous study [24], 96% of all desaturations were shorter, and only 4.0% equal to, or longer than, 20 s (using a averaging time of 3 s).

In our study, the number of desaturations of duration ≥20 s was also influenced by averaging time, but, in contrast to short events, the slope was positive, i.e. more desaturations lasting ≥20 s were counted with a longer averaging time. In this case, for instance, due to the smoothing effect of longer averaging times, two desaturations with a duration of 15 s could have been summed up to one desaturation ≥20 s, which means, that the number of severe events would have been increased.

Based on the data reported here, what would be the most appropriate averaging time? This will depend on the purpose of monitoring desaturation events. In an intensive care setting, where staff should not be desensitized from responding to a clinically meaningful alarm by too many prior alarms resulting from short, self resolving events, a longer averaging time (e.g., 10 s) may be preferable. For recordings of SpO_2_ in a sleep study, however, where knowledge about short desaturations potentially eliciting frequent arousals is relevant, a short averaging time (e.g., 3 s) may be preferable.

For desaturation events with a minimal duration of 10–15 s, the averaging time has almost no influence, indicated by exponents *c* close to 0 for a minimal duration of 10 s. Only for such desaturation events may the influence of the averaging time be ignored.

Are there any prior studies to demonstrate the relevance of our formula? A recent crossover study compared low-flow air or oxygen via nasal cannulae to prevent desaturation in preterm infants. Infants had significantly less intermittent desaturations to <80% SpO_2_ while receiving oxygen (4 vs. 18) [38]. Unfortunately no mention was made on the averaging time of the oximeter, thus there are difficulties in translating these results into other neonatologists' daily practice (e.g., were these relevant desaturations?).

Barker et al. tested 20 different oximeters simultaneously under standardized conditions to analyze the influence of motion on saturation values. All oximeters were reported to have been tested in their default mode, but this may vary: the default mode may be 12 seconds for the 3740 and 3800 instrument, but 10 seconds for the AS/3 (all Datex-Ohmeda, Helsinki, Finland) [4]. According to the authors, the oximeters' ability to detect hypoxemic events was quantified by specificity and sensitivity, where sensitivity was defined as the proportion of time the test oximeter read <90% when the SpO_2_ was also <90% [4]. How reliable can the resulting ROC curves be calculated without taking the different averaging times used into account? Here, our formula may help to adjust the data on number of desaturations seen with the different oximeters.

Heimann et al. described the impact of skin to skin care and positioning on cardiorespiratory parameters and thermoregulation in premature infants. Episodes of desaturation to <80% were significantly more frequent in supine position compared to prone position. They used an oximeter with an averaging time of 4 s [39]. If another NICU uses an averaging time of, e.g., 10 s, our formula may help to estimate whether the differences reported here would still be valid with a much longer averaging time.

One of the authors reported reference values for pulse oximetry recordings in healthy term neonates during their first 5 days of life [40]. The maximum value for the desaturation event rate (desaturations per hour) in their study was at 47.1 for desaturations to <90% and at 15.0 for those <85%, based on an averaging time of 2–4 s and a desaturation duration ≥1 s. These values would have been reduced to 16.4 and 4.8 had an averaging time of 10 s been used (See Table 4). Thus, units basing treatment decisions for apnea of prematurity on a specific threshold for the rate of desaturations may utilize our formula to adapt their own upper limits of normal to the instrument settings used in their patients.

**Table 4:** Reference values for pulse oximetry recordings in healthy term neonates during their first 5 days of life for 7 different averaging times and three SpO_2_ thresholds.

SpO_2_		Based on ‘SIQ’ definition				Based on ‘SIQ & PW’ definition			
Threshold (%)	T (sec)	Median	75th Centile	95th Centile	Maximum	Median	75th Centile	95th Centile	Maximum
**90**	3	**1.78**	**4.61**	**15.52**	**47.14**	**0.78**	**2.50**	**8.38**	**36.43**
	5	1.14	2.95	9.92	30.14	0.50	1.60	5.36	23.29
	8	0.75	1.95	6.57	19.97	0.33	1.06	3.55	15.43
	10	0.62	1.61	5.41	16.42	0.27	0.87	2.92	12.69
	12	0.53	1.37	4.61	14.00	0.23	0.74	2.49	10.82
	14	0.46	1.20	4.03	12.23	0.20	0.65	2.17	9.45
	16	0.41	1.06	3.58	10.88	0.18	0.58	1.93	8.41
**85**	3	**0.00**	**0.42**	**1.74**	**15.00**	**0.00**	**0.21**	**1.07**	**11.19**
	5	0.00	0.25	1.07	9.22	0.00	0.13	0.66	6.88
	8	0.00	0.16	0.68	5.89	0.00	0.08	0.42	4.39
	10	0.00	0.13	0.55	4.76	0.00	0.07	0.34	3.55
	12	0.00	0.11	0.46	4.00	0.00	0.06	0.29	2.98
	14	0.00	0.10	0.40	3.45	0.00	0.05	0.25	2.58
	16	0.00	0.08	0.35	3.04	0.00	0.04	0.22	2.27
**80**	3	**0.00**	**0.00**	**0.54**	**2.32**	**0.00**	**0.00**	**0.39**	**1.74**
	5	0.00	0.00	0.31	1.34	0.00	0.00	0.22	1.00
	8	0.00	0.00	0.19	0.80	0.00	0.00	0.14	0.60
	10	0.00	0.00	0.15	0.63	0.00	0.00	0.11	0.47
	12	0.00	0.00	0.12	0.52	0.00	0.00	0.09	0.39
	14	0.00	0.00	0.10	0.44	0.00	0.00	0.07	0.33
	16	0.00	0.00	0.09	0.38	0.00	0.00	0.06	0.28

The basic values are based on a clinical study where an averaging time of 2–4 s has been used for desaturations with an arbitrary minimal duration. When applying our formula the reference values for 6 other averaging times could be calculated (SIQ: signal quality indicator; PW: pulse waveform; SpO2: pulse oximeter saturation; T (sec): Averaging time in seconds.

It seems clear from Figure 2 that the infant in question had three rather prolonged episodes of arterial desaturation – first to the mid-80s, second to the high 70s, and third to the low 80s. This presentation of SpO_2_ might be much more important for clinical decision making than simply knowing a larger number of short desaturation episodes. With other words, not only the threshold of desaturation has to be defined, but also their duration. In some studies, the desaturation duration has not been taken into account, so that a minimal duration >0 s may be assumed. In other cases, an event with a desaturation duration of ≥20 s has been defined as severe [31]. We took into account that the desaturation duration plays an important role and therefore integrated the duration into our formula. The formula can be applied for different desatuaration durations, the only thing one has to do is to use the appropriate c value, which can be found in table 2.

Limitations of our study include the fact that we used only one oximeter brand (Masimo, Radical). In future studies other oximeters should be used for this type of analysis, specially, if different algorithm for calculating the averaging time are used. Unfortunately, the manufacturer does not provide information about the algorithm that is used for smoothing the SpO_2_ time series. Taking this fact into account, generalizability of our formula to other oximeters remains limited. In further studies, parallel to the reprocessed data, “real” observed data should be analyzed by using two oximeters simultaneously. However, such an approach may generate new problems, as oximeters never measure exactly the same values because of motion or other artifacts [3]–[8].

Another limitation is that – as mentioned in the Methods section - the manufacturer uses an algorithm with a moving average for short averaging times (2–4 s and 4–6 s). This problem might be mitigated by the fact that the differences regarding the number, depth and duration of desaturations comparing short to long averaging times (e.g., 16 s) are so large that the influence of the moving average at 2–4 and 4–6 s can most probably be ignored. With regard to the wide range of SpO_2_ measurements (at least in intensive care, sleep medicine and neonatology), our analyses refer to desaturations in premature infants, which means that further research is necessary to evaluate whether our formula can be applied equally to children and adults. In the future, it would also be desirable to generate a formula for other desaturation definitions, e.g. by a decline of ≥3%, which is often used in sleep medicine[30], instead of counting the numbers of desaturations below a fixed threshold (e.g. <85%).
